# Curved reformat of the paediatric brain MRI into a ‘flat-earth map’ — standardised method for demonstrating cortical surface atrophy resulting from hypoxic–ischaemic encephalopathy

**DOI:** 10.1007/s00247-016-3638-3

**Published:** 2016-06-23

**Authors:** Ewan Simpson, Savvas Andronikou, Schadie Vedajallam, Anith Chacko, Ngoc Jade Thai

**Affiliations:** 1 Department of Pediatric Radiology, Bristol Royal Hospital for Children, Upper Maudlin Street, Bristol, BS2 8BJ UK; 2 CRICBristol, University of Bristol, Bristol, UK

**Keywords:** Children, Cortex, Curved reformat, Hypoxic–ischaemic encephalopathy, Magnetic resonance imaging, Watershed

## Abstract

Hypoxic–ischaemic encephalopathy is optimally imaged with brain MRI in the neonatal period. However neuroimaging is often also performed later in childhood (e.g., when parents seek compensation in cases of alleged birth asphyxia). We describe a standardised technique for creating two curved reconstructions of the cortical surface to show the characteristic surface changes of hypoxic–ischaemic encephalopathy in children imaged after the neonatal period. The technique was applied for 10 cases of hypoxic–ischaemic encephalopathy and also for age-matched healthy children to assess the visibility of characteristic features of hypoxic–ischaemic encephalopathy. In the abnormal brains, fissural or sulcal widening was seen in all cases and ulegyria was identifiable in 7/10. These images could be used as a visual aid for communicating MRI findings to clinicians and other interested parties.

## Introduction

MRI of the brain in term neonates with hypoxic–ischaemic encephalopathy reveals damage to the cerebral cortex with characteristic morphology (i.e. ulegyria) in characteristic locations, depending on the severity and duration of the insult [1]. Communicating the bilateral symmetrical geographic involvement of the brain to parents and the legal fraternity contesting compensation rights using standard cross-sectional images is challenging. An overview map of the whole brain surface generated from a curved reconstruction of the MR images may be useful for demonstrating the damage in such scenarios.

Previous work on curved reconstruction of the brain surface centred around demonstrating focal cortical lesions [2, 3]. We hypothesised that the characteristic regional cortical atrophy seen in hypoxic–ischaemic encephalopathy might be well-demonstrated using this method. This paper describes a standardised method of generating curved reconstruction of the paediatric brain from 3-D MRI in order to demonstrate the surface atrophy of the cerebral hemispheres in children with who sustained hypoxic–ischaemic encephalopathy at term delivery. We have dubbed the resulting images “flat-earth maps”. By comparing 10 children who sustained hypoxic–ischaemic encephalopathy at term delivery with age-matched normal cases, we demonstrate that the characteristic findings of hypoxic–ischaemic encephalopathy are visible on these maps.

## Description

We employed the OsiriX freeware (Pixmeo SARL, Bernex, Switzerland) image-viewing platform to generate a standardised method of producing curved reconstructions of the paediatric brain.

MRI scans of 10 children with known cortical atrophy caused by hypoxic–ischaemic encephalopathy sustained during term delivery were selected alongside 8 age-matched controls who had normal MRI findings. The research was approved by the University of Bristol Ethics Committee (case reference 27741) and has been performed in accordance with the ethics standards laid down in the 1964 Declaration of Helsinki and its later amendments.

We selected data from T1-weighted 3-D turbo spin-echo or 3-D fluid-attenuated inversion recovery (FLAIR) imaging. Using the 3-D curved MPR function in OsiriX, multiple curved reformatting techniques were tested, then formalised and standardised. We performed reconstructions from all three planes (coronal, sagittal and axial) using different landmark slices (e.g., foramen of Monro, pineal gland), angles of reconstruction (e.g., following the course of the Sylvian fissure, central sulcus) and at various depths to the cortical surface.

Two images (one reconstructed from the coronal plane and one from the sagittal plane) were determined to give the best overview of the cortical regions most frequently damaged in hypoxic–ischaemic encephalopathy sustained at term delivery (i.e. the watershed zones, and perisylvian and perirolandic regions). We describe an optimal method for creating these reconstructions.

**Fig. 1:**
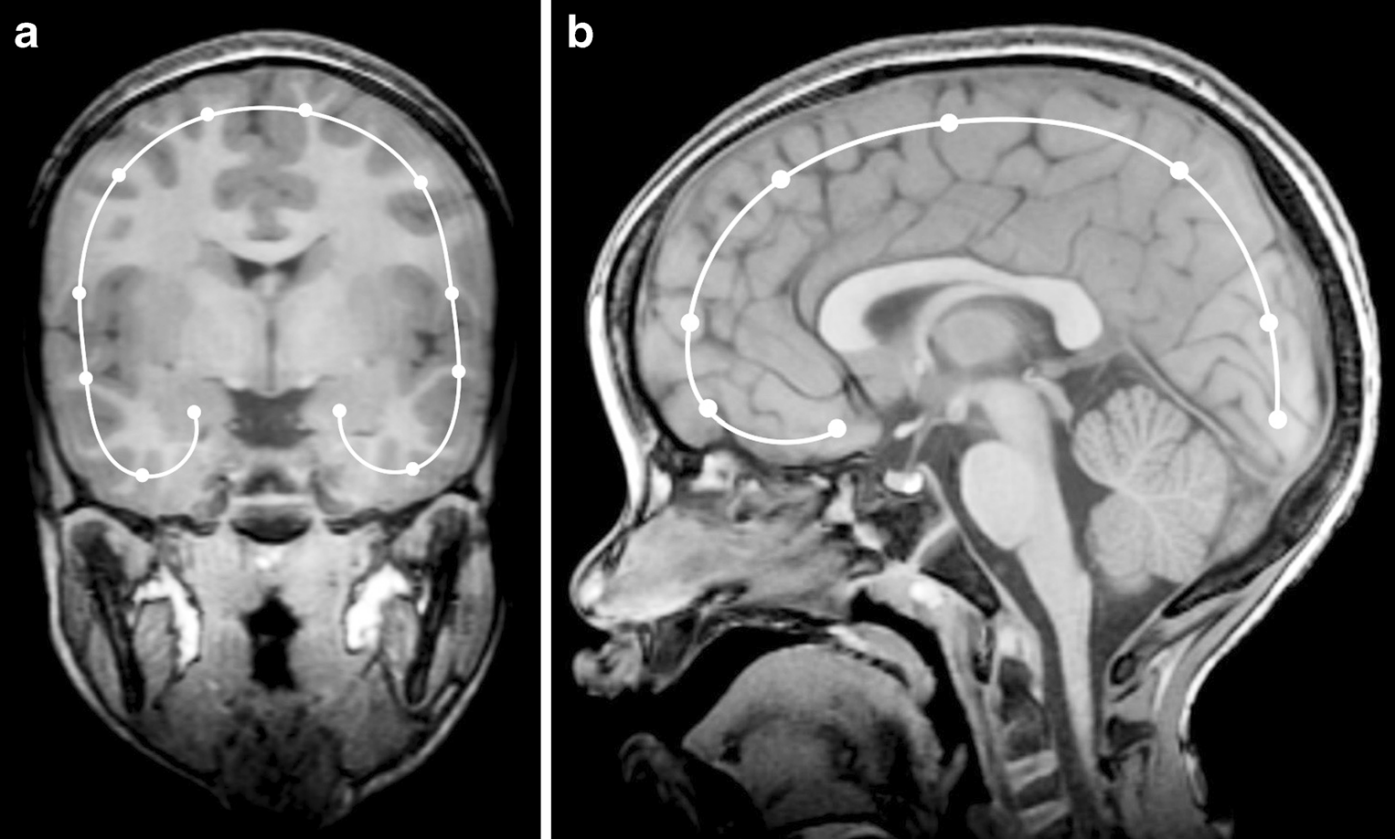
T1-weighted 3-D turbo spin-echo brain MRI in a 7-year-old boy in the control group is used to show deposited cursors for generating flat-earth maps. **a** The landmark coronally reconstructed image for generating a Mercator map is the one demonstrating the foramina of Monro. The pathway for generating the curved reconstructions is plotted 1-cm deep to the surface of the brain by depositing cursors at the following 6 landmarks in a clockwise direction on both sides of the midline (a total of 12 points): lateral hippocampus, inferior temporal gyrus, gyrus inferior to Sylvian fissure, gyrus superior to Sylvian fissure, gyrus midway from the previous to the final point, gyrus abutting the midline/falx. **b** The landmark midline sagittal reconstructed image demonstrating the aqueduct of Sylvius is selected for generating a scroll map. The pathway for generating the curved reconstructions is plotted 1-cm deep to the cortical surface by depositing cursors at 8 points starting anteriorly and ending posteriorly: posterior aspect of the straight gyri (1 cm anterior to the optic chiasm), the anterior aspect of the straight gyri, anterior frontal lobes along a horizontal line extending through the genu and splenium of the corpus callosum, halfway between the previous and next points, posterior frontal lobes along a vertical line extending from the upper cervical cord and brainstem, halfway between the previous and next points, occipital lobes along a horizontal line extending through the genu and splenium of the corpus callosum, posterior occipital lobe 1 cm superior and anterior to the torcula. The resultant image derived from sagittal images is likened to unrolling a scroll

For reconstructions from the coronal 3-D T1-weighted data set, the slice demonstrating the foramina of Monro is selected for generating the flat-earth map. The pathway for generating the curved reconstructions is plotted 1-cm deep to the surface of the brain by depositing cursors at 12 landmarks (6 on either side of the midline) as described in Fig. 1. The resultant image has been likened to a Mercator map of the earth [3, 4].

For reconstructions from the sagittal 3-D T1-weighted data, the landmark midline slice demonstrating the aqueduct of Sylvius is selected. The pathway for generating the curved reconstructions is plotted 1-cm deep to the cortical surface by depositing cursors at 8 points starting anteriorly and ending posteriorly as described in Fig. 1. The resultant image derived from sagittal images is likened to unrolling a scroll.

**Fig. 2:**
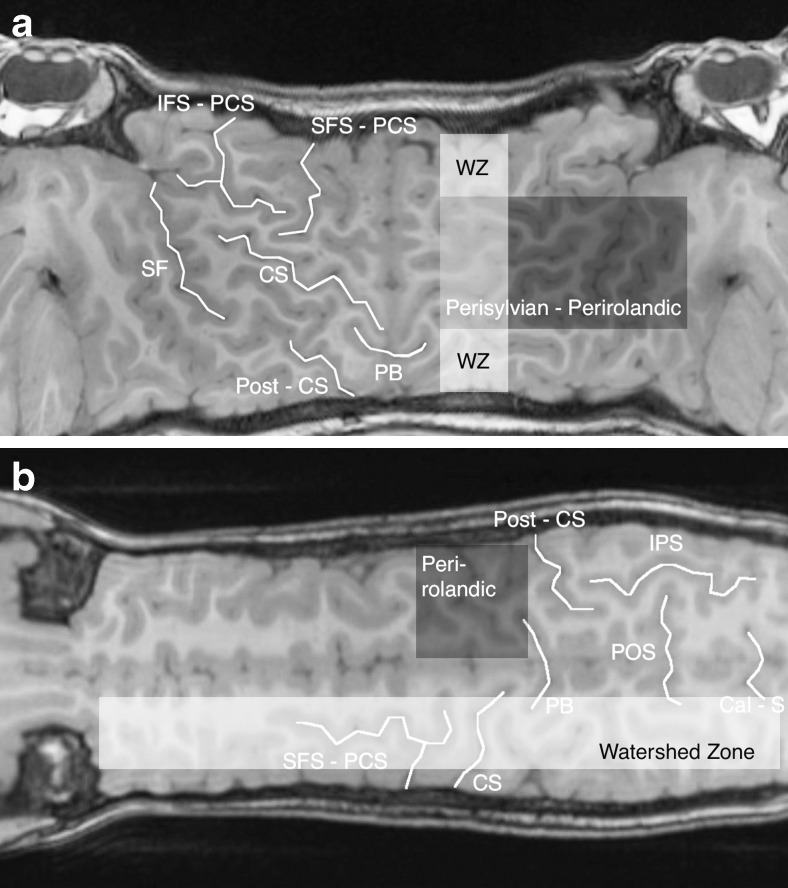
Mercator and scroll maps generated from the T1-weighted 3-D turbo spin-echo brain MRI in a 7-year-old boy in the control group demonstrate sulcal and fissural anatomy for the purposes of defining the watershed and perirolandic regions involved in hypoxic–ischaemic encephalopathy. **a** Mercator map reconstructed from coronal images demonstrates the inferior frontal sulcus–precentral sulcus sign (*IFS*-*PCS*), superior frontal sulcus–precentral sulcus sign (*SFS*-*PCS*), central sulcus (*CS*), Sylvian fissure (*SF*), pars bracket (*PB*) and postcentral sulcus (*post*-*CS*). In addition, the watershed region (*WZ*) and perirolandic/perisylvian continuum is indicated in shaded blocks, based on the identified anatomy. **b** Scroll map reconstructed from sagittal images demonstrates the superior frontal sulcus joining the precentral sulcus (*SFS*-*PCS*), central sulcus (*CS*), pars bracket (*PB*), postcentral sulcus (*post*-*CS*), parieto-occipital sulcus (*POS*), intraparietal sulcus (*IPS*) and calcarine sulcus (*Cal*–*S*). In addition, the watershed zone and perirolandic regions are indicated in shaded blocks based on the identified anatomy

The cerebral sulcal anatomy of children in the control group from the Mercator brain maps was identified using work by Wagner et al. [4]. No equivalent anatomical work exists for scroll brain maps, and the sulcal anatomy was identified de novo from an online anatomy atlas [5]. The sulcal anatomy and expected regions of abnormality were then plotted on the Mercator and scroll maps in one of the control patients as a reference source (Fig. 2).

Flat-earth maps (Mercator and scroll projections) of the brain were then generated for 10 children with previous hypoxic–ischaemic encephalopathy. These were displayed alongside those of age-matched healthy controls for comparison. Two readers (S.A., a paediatric radiologist with 15 years’ experience in neuroimaging, and E.S., a 5th-year radiology trainee) assessed each flat-earth map for the presence of (1) widening of the Sylvian fissure; (2) widening of the longitudinal fissure, noting whether such widening was localised (indicating regional watershed-zone, or perisylvian or perirolandic volume loss) or generalised; (3) cortical atrophy in characteristic locations (perirolandic, perisylvian or watershed distribution), and (4) ulegyria. Discrepancies were arbitrated by a third reader (A.C., a qualified general radiologist 3 years post completion of training).

### Results

No abnormal features were identified on any of the eight controls (mean age 3 years 11 months; 5 boys, 3 girls). The abnormal findings that were visible in children with hypoxic–ischaemic encephalopathy sustained at term delivery (mean age 3 years 6 months; 6 boys, 4 girls) are shown in Table 1.

**Table 1:** Regional volume loss and presence of ulegyria as detected from the flat-earth maps in 10 children who sustained perinatal hypoxic–ischaemic injury at term delivery

Case	Age	Sex	Widening of Sylvian fissure	Widening of longitudinal fissure		Regional atrophy			Ulegyria
				Overall	Localised lentiform	Rolandic	Sylvian	Watershed	
2	2 y 1 m	M	Y	Y	Y	Y	Y	Y	Y
6	5 y 11 m	M	Y	Y	Y	Y	Y	Y	Y
8	2 y 10 m	F	Y	Y	Y	Y	Y	Y	N
10	4 y 10 m	F	Y	Y	N	Y	N	Y	Y
15	7 y	M	Y	Y	Y	Y	Y	Y	Y
18	2 y	F	Y	Y	Y	Y	Y	Y	N
20	4 y	M	Y	Y	Y	Y	Y	Y	Y
21	3 y 10 m	M	Y	Y	Y	Y	Y	Y	Y
24	1 y 10 m	F	Y	Y	Y	N	Y	Y	Y
27	1 y 4 m	M	Y	Y	Y	Y	Y	Y	N
Total	3 y 6 m (mean)	6 M, 4 F	10 (100%)	10 (100%)	9 (90%)	9 (90%)	9 (90%)	10 (100%)	7 (70%)

*F* female, *M* male, *m* months, *N* no, *y* years, *Y* yes

The flat-earth map generated by the curved reconstruction displays the structures along a region adjacent to the plotted path at the expense of structures at the extremes of the axis perpendicular to that path; two maps are generated from perpendicular curved lines, so different structures are seen optimally on each map. The following general observations were made:

- The perisylvian and perirolandic regions were better demonstrated on the Mercator maps. The scroll maps demonstrated the lateral structures poorly.
- More of the frontal lobe anatomy was seen consistently on the Mercator maps.
- The central posterior parietal and occipital lobes were better demonstrated on the scroll maps.
- Watershed zones were well demonstrated on both Mercator and scroll maps.

**Fig. 3:**
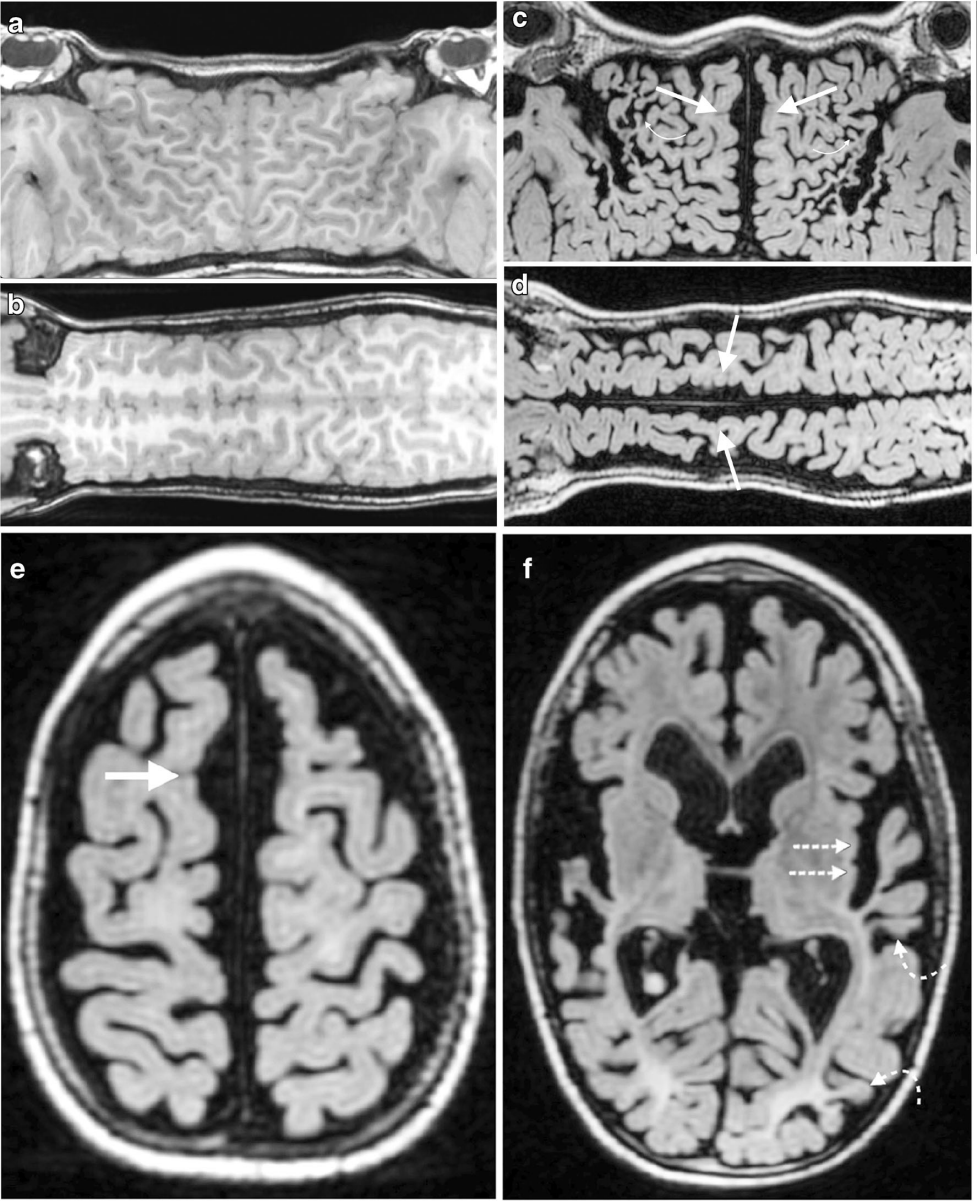
Comparison of normal and abnormal T1-weighted 3-D turbo spin-echo brain MRI using the suggested post-processing technique. **a**, **b** Images in a 7-year-old boy from the control group. **a** Mercator map reconstructed from the coronal images in the control child demonstrates the parallel hemispheres, which are abutting at the midline, and also demonstrates the close proximity of the frontal and temporal lobes to each other at the Sylvian fissures. **b** Scroll map reconstructed from the sagittal images in the control child demonstrates the normal gyral anatomy with parallel hemispheres in close proximity to each other at the midline. **c**–**f** Images in a 7-year-old boy who sustained hypoxic–ischaemic injury at term delivery. **c** Mercator map demonstrates bilateral parafalcine atrophy separating the hemispheres from the midline in a bi-convex or lentiform manner (*straight arrows*) as well as perisylvian atrophy with visible ulegyria (*curved arrows*). The ulegyria is manifest as gyri with preferential thinning at the base as opposed to the apex, resulting in a mushroom-shape or drumstick appearance. **d** Scroll map demonstrates bilateral parafalcine atrophy separating the hemispheres from the midline in a bi-convex or lentiform manner (*arrows*). **e** Standard axial slice demonstrates parafalcine atrophy in the frontal lobes (*arrow*), which correlates well with the Mercator and scroll projections. **f** Standard axial slice demonstrates the perisylvian atrophy (*straight arrows*) and ulegyria (*curved arrows*), in both the perisylvian inter-vascular watershed regions

**Fig. 4:**
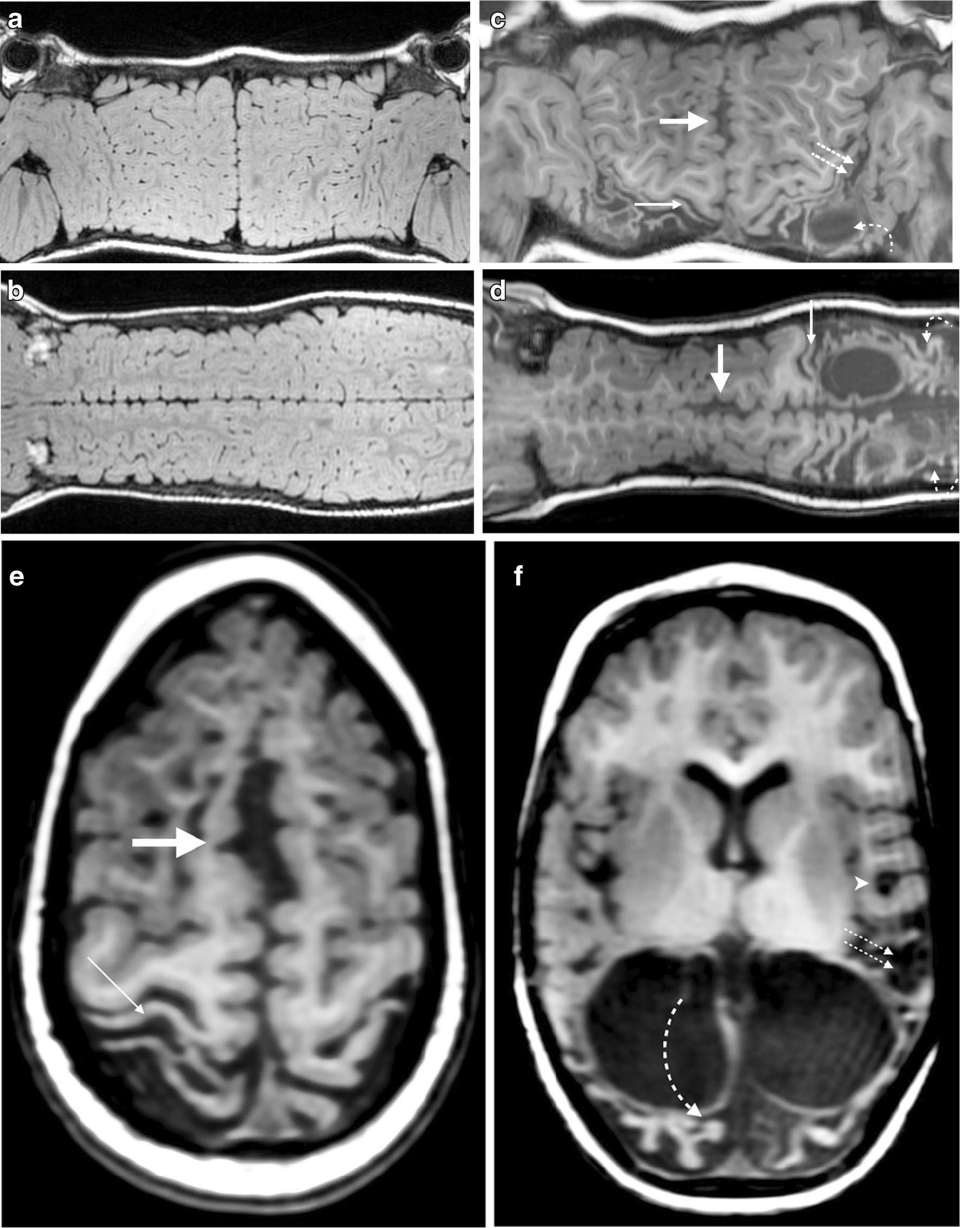
Comparison of normal and abnormal T1-weighted 3-D turbo spin-echo brain MRI using the suggested post-processing technique. **a**, **b** Images in a 3-year-old male from the control group. **a** Mercator map reconstructed from the coronal images in the control child demonstrates the parallel hemispheres, which are abutting at the midline, and also demonstrates the close proximity of the frontal and temporal lobes to each other at the Sylvian fissures. **b** Scroll map reconstructed from the sagittal images in the control child demonstrates the normal gyral anatomy with parallel hemispheres in close proximity to each other at the midline. **c**–**f** Corresponding images from a 3-year-old female who sustained hypoxic–ischaemic injury at term delivery. **c** Mercator map demonstrates parafalcine atrophy, separating the hemispheres from the midline in a lentiform manner (*thick arrow*). This map shows widening of the Sylvian fissures (*straight dotted arrows*) and marked atrophy of the post-central gyrus (*thin arrow*). There is evidence of posterior inter-vascular watershed damage with cystic change (*curved arrow*), but the extent is not as well demonstrated as with the scroll map. **d** Scroll map demonstrates parafalcine atrophy, separating the hemispheres from the midline in a lentiform manner (*thick arrow*). This map shows marked atrophy of the post-central gyrus (*thin arrow*) and of the posterior inter-vascular watershed regions (*curved arrows*). There are ulegyria and cystic changes in this region, which is shown to better effect than on the Mercator map. **e** Standard axial image through the vertex demonstrates the parafalcine atrophy causing lentiform separation of the hemispheres (*thick arrow*) and the perirolandic atrophy (*thin arrow*), which correlate well with the appearances on the Mercator and scroll maps. **f** Axial slice at the level of the lateral ventricles demonstrates extensive posterior watershed distribution atrophy, which correlates with the scroll map (**d**) and to a lesser extent with the Mercator map (**c**). There is visible ulegyria in the occipital lobes (*curved arrow*) while tear-drop-shape sulci are confirmatory features of ulegyria in the left temporal lobe (*arrowhead*). Widening of the Sylvian fissure is noted (*straight arrows*) and correlates well with the appearances on the Mercator map

The following observations were made from review of flat-earth maps in children who sustained hypoxic–ischaemic injury at term delivery:

- Widening of the longitudinal fissure was seen on all abnormal cases on Mercator and scroll maps, consistent with parasagittal/parafalcine watershed atrophy. In 9 of 10 instances there was a localised biconvex separation of the hemispheres distinct from the parallel separation of the hemispheres elsewhere (Figs. 3 and 4).
- Damage to intervascular watershed zones was well seen on both Mercator and scroll maps (anterior watershed zones were better demonstrated on Mercator map and posterior watershed regions on the scroll maps) and was noted in a continuous band-like fashion (as opposed to a wedge of abnormality) (Fig. 4).
- Perirolandic and perisylvian damage was better demonstrated on Mercator maps (Figs. 3 and 4).
- Ulegyria was identified in 7 of 10 of cases of hypoxic–ischaemic encephalopathy (Figs. 3 and 4, Table 1).

## Discussion

### Perinatal hypoxic–ischaemic encephalopathy

Characteristic cortical abnormalities in hypoxic–ischaemic encephalopathy sustained at term delivery depend on both the severity and duration of the hypoxic–ischaemic insult [1]. MRI is ideally performed in the first 10 days of age, but work in the United Kingdom has shown that adherence to such imaging protocols is suboptimal [6]. Delayed imaging is therefore often performed and demonstrates atrophy of the affected regions. In acute near-total asphyxia at term, cortical abnormality is seen in the perirolandic regions, in addition to the ventrolateral thalami and posterior putamina. Partial prolonged asphyxia in term infants results in volume loss with ulegyria (mushroom-shape gyri) in the parasagittal and other watershed zones of perfusion [1]. These global insults result in damage that is bilateral and symmetrical but not always of equal severity on each side. The two flat-earth maps that we have described give an excellent overview of the regions of cortex that are most commonly affected in hypoxic–ischaemic encephalopathy, allowing the resultant bilateral multifocal atrophy and characteristic ulegyria to be visualised simultaneously.

### Curved reformatting of the brain

A method for improved display of brain surface anatomy using curved reconstructions of MRI was described by Bastos et al. [2] in 1995; these authors demonstrated focal cortical dysgenesis better than can be seen on standard multiplanar imaging. Subsequent publications have used the Mercator view to depict surface lesions [3] and sulcal patterns to define gyral anatomy [4]. To our knowledge, ours is the first paper to use curved reconstruction of MRI to display bilateral cortical regional atrophy to advantage.

There are other methods of creating curved reconstructions on the brain surface, some of which are automated and therefore do not require points to be set manually [7]. However these techniques sometimes require dedicated software that is costly to purchase and requires extensive training to use. Our technique can be performed using software bundled with commercially available MRI scanners. It has the advantages of being free, easily taught and quick to perform, each curved reconstruction taking less than a minute to generate. The disadvantage of our method is that no single flat-earth map can demonstrate the entire cortex optimally. Methods that generate 3-D displays of curved reconstruction require image-viewing software (because the brain is presented as a 3-D surface, which must be virtually rotated to view from all angles), but these methods can display the entire brain and therefore merit further research for displaying the cortical changes of hypoxic–ischaemic encephalopathy.

### Utility of the flat-earth map for non-radiologists

Several groups of people have a professional or personal interest in the radiology report in cases of hypoxic–ischaemic encephalopathy. Clinicians who require information on neuroimaging include hospital providers such as neonatologists and neurologists, who usually have access to the MR imaging, but also general practitioners in the community, who may not. The parents of children who have sustained brain injury might also value access to the radiology report, but it is known that patients can find it difficult to understand the complex terminology prevalent in radiology reports [8]. Recently Sadigh et al. [9] surveyed more than 200 referring clinicians and found that most would be more likely to discuss a radiology report with patients — and provide them with a copy — if embedded images were available. Embedding the flat-earth map into a report might represent an elegant solution (without the need to provide selected image slices in a variety of planes) to discuss the MRI findings with parents. Furthermore, neuroimaging is now routinely submitted as evidence in the courtroom, and flat-earth images could therefore be used to support allegations of perinatal asphyxia [10]. The flat-earth maps could provide an overview of the brain surface to demonstrate the extent of bilateral regional cortical damage in a way that single slices from an orthogonal plane could not.

In conclusion, a standardised method of curved reconstruction of the brain surface from 3-D MRI allows visualization of key regions of cortical atrophy in cases of term hypoxic–ischaemic encephalopathy. This visualisation takes the form of two flat-earth maps: Mercator and scroll. Each of these maps displays different parts of the cortex to good effect and is complementary to the other. These maps negate the need for viewing the multitude of MRI slices in three planes when communicating multifocal, bilateral cortical regional atrophy to non-medical specialists such as legal professionals and parents, and they can also be easily embedded within a radiology report.
